# The intellectual challenges and emotional consequences of equipoise contributed to the fragility of recruitment in six randomized controlled trials^☆^

**DOI:** 10.1016/j.jclinepi.2014.03.010

**Published:** 2014-08

**Authors:** Jenny L. Donovan, Isabel de Salis, Merran Toerien, Sangeetha Paramasivan, Freddie C. Hamdy, Jane M. Blazeby

**Affiliations:** a School of Social and Community Medicine, University of Bristol, Canynge Hall, 39 Whatley Road, Bristol BS8 2PS, UK; b Department of Sociology, Wentworth College, University of York, Heslington, York YO10 5DD, UK; c Nuffield Department of Surgical Sciences, University of Oxford, Old Road Campus Research Building, Oxford OX3 7DQ, UK

**Keywords:** Randomized controlled trials, Recruitment, Equipoise, Uncertainty, Qualitative research, Uncertainty, Training

## Abstract

**Objective:**

The aim of the study was to investigate how doctors considered and experienced the concept of equipoise while recruiting patients to randomized controlled trials (RCTs).

**Study Design and Setting:**

In-depth interviews with 32 doctors in six publicly funded pragmatic RCTs explored their perceptions of equipoise as they undertook RCT recruitment. The RCTs varied in size, duration, type of complex intervention, and clinical specialties. Interview data were analyzed using qualitative content and thematic analytical methods derived from grounded theory and synthesized across six RCTs.

**Results:**

All six RCTs suffered from poor recruitment. Doctors wanted to gather robust evidence but experienced considerable discomfort and emotion in relation to their clinical instincts and concerns about patient eligibility and safety. Although they relied on a sense of community equipoise to justify participation, most acknowledged having “hunches” about particular treatments and patients, some of which undermined recruitment. Surgeons experienced these issues most intensely. Training and support promoted greater confidence in equipoise and improved engagement and recruitment.

**Conclusion:**

Recruitment to RCTs is a fragile process and difficult for doctors intellectually and emotionally. Training and support can enable most doctors to become comfortable with key RCT concepts including equipoise, uncertainty, patient eligibility, and randomization, promoting a more resilient recruitment process in partnership with patients.

**What is new?**

• Poor recruitment threatens the validity of randomized controlled trials (RCTs) and their power to answer key health-care questions and wastes research resources, and this study has illuminated, from the recruiter's perspective, why it is such a fragile process.
• Doctors in these RCTs were keen to participate in RCTs to gather high-quality evidence, but they struggled intellectually and emotionally with the conflicts between the needs of the RCTs and their clinical instincts, treatment preferences, and concerns about patient eligibility and safety.
• Equipoise was relatively easy for doctors to define in general terms and particularly as uncertainty among a community of experts, but it became a much more elusive concept when they attempted to recruit patients with particular clinical characteristics to the RCT.
• Doctors need training and support to enable them to become more familiar and comfortable with key RCT concepts including equipoise so that they can engage more easily with patients and participate in a more resilient recruitment process.

## 1 Introduction

Recruitment to randomized controlled trials (RCTs) is difficult. Approximately 50% of initiated RCTs reach their original recruitment target [1], and poor recruitment undermines the power of RCTs to answer key questions and their external validity and leads to considerable waste of research resources. Most research on RCT recruitment has focused on ways of increasing patient participation, for example, by providing additional or favorable information [2] or comparing lengths of information sheets [3]. However, systematic reviews have identified only a small number of successful interventions directed at patients [4] and pointed to the lack of prospective research in ongoing RCTs and studies involving recruiters [5]. The research that has been done with recruiters includes a survey of pediatricians that suggested that their views could influence levels of participation [6] and two studies that have indicated that difficulties with equipoise can act as a barrier to recruitment to RCTs [7,8].

There has been considerable debate about the concept of “equipoise” in relation to RCTs since the study design was first formally recognized in the 1940s. At the heart of the debate is the need to justify the recruitment of patients to studies incorporating an experiment rather than ensuring they receive the best medical care under a physician's guidance. Bradford Hill [9] established the principle that a doctor should only include a patient in an RCT if they could claim “no knowledge that one treatment will be better or worse, safer or more dangerous than another.” The term equipoise was later defined by Fried [10], “where the balance of opinion is truly in equipoise, there is no sense to the accusation that the prescribing of the one or the other of the equally eligible treatments can constitute a withholding of anything or can constitute doing less than one's best.”

Over time and with the growth in numbers of RCTs carried out, there has been a shift toward an acknowledgment that the conduct of RCTs could be justified by a lack of satisfactory evidence and consensus among experts about the comparative merits of treatments [11]. In the late 1990s, the concept of “uncertainty” was then advocated—that an RCT could be undertaken when doctors were uncertain [12] or “substantially” uncertain [13], or there was consensus about uncertainty among a community of doctors [14]. In more recent years, the debate has become polarized between those strongly advocating the need for the concept of equipoise to provide legal and ethical justification for RCTs [15,16] and others rejecting it as unnecessary and impossible to operationalize [17,18].

A research program was undertaken to understand the recruitment process in pragmatic RCTs experiencing, or expecting to experience, poor recruitment and, where possible, to provide feedback to improve recruitment [15]. Interviews undertaken with doctors from different specialties focused on how the concepts of equipoise and uncertainty were used in everyday practice and revealed the practical and emotional consequences as doctors struggled to reconcile the conflicts they experienced in their clinical and research roles. Training and support needs were identified for doctors to help them negotiate the fragile process of RCT recruitment with patients.

## 2 Study design and setting

RCTs that were experiencing, or expecting to experience, poor recruitment were identified through contact with funding bodies and informal communication with trialists. The chief investigators (CIs) of seven RCTs were approached and asked to participate in an integrated qualitative study of recruitment [19]. The CIs were asked to be interviewed and to identify key RCT personnel and staff undertaking recruitment in the RCTs who could be approached to be interviewed about recruitment. All seven CIs agreed to participate; in one RCT, recruitment started well, and so the qualitative research was not undertaken. The six RCTs included were pilot/feasibility studies, pragmatic in design, funded by national bodies, and encompassed a range of clinical contexts, different types of intervention, and types of recruitment staff (Table 1). Ethical approval was obtained within the governance arrangements of each RCT. Interviews were undertaken with the CI and doctors actively involved in recruitment who agreed to participate in the qualitative research. Consent was obtained for interviews to be audio recorded. Views about equipoise were sought through in-depth interviews using a checklist of topics to ensure that similar issues were covered with participants while enabling other issues of importance to emerge. Interviews were conducted between 2001 and 2009 by Zelda Tomlin, S.P., I.d.S., M.T., or Gavin Daker-White, mostly face-to-face or by telephone when participants were difficult to reach, with some in groups when recruiters met for study purposes. In four RCTs, follow-up interviews were carried out with 15 recruiters (Table 1).

Interviews were transcribed in full, and data were analyzed using qualitative content and thematic analysis methods, based on the techniques of constant comparison and grounded theory [26,27]. Data relating to uncertainty and equipoise were extracted from the original interview transcripts and systematically coded, analyzed, and synthesized by J.L.D. in 2011–2013 to identify similarities and differences across the RCTs in how the concepts were considered and experienced and to investigate implications for recruitment and training needs.

## 3 Results

The six pragmatic RCTs varied in terms of size and duration and encompassed a range of clinical specialties and complex interventions (Table 1). In each RCT, the CI and specialist hospital doctors, general practitioners, nurses, and other clinical staff conducting recruitment were interviewed (72 in total). The focus of this analysis was on doctors in clinical practice who were actively involved in recruitment and for whom equipoise was most relevant—32 in total, 15 of whom were interviewed on more than one occasion (Table 1). There were 21 surgeons and 11 specialists from primary care, oncology, or psychiatry. All doctors readily discussed their involvement in the RCTs, the challenges of recruitment, and issues related to equipoise or uncertainty. In the presentation of findings below, quotations have been provided to support the analysis, some of which are included in the Appendix at www.jclinepi.com. Interviewee details have been anonymized to protect confidentiality and labels used to indicate the doctor and RCT (eg, T8-S1 (RCT8 surgeon 1) or T8-D2 (RCT8 other specialist 2)) and whether the interview was in a group (-G) or follow-up (-F).

### 3.1 Individual or community equipoise

Some doctors defined themselves as being in “individual” equipoise—convinced that RCT treatments were suitable for eligible patients, but most relied on a sense of “community” equipoise arising from a lack of evidence and consensus among colleagues about the best treatment (Box 1).

### 3.2 Difficulties and discomfort in relation to equipoise

As the doctors discussed equipoise and their roles and responsibilities in relation to RCT recruitment and clinical practice, they expressed considerable discomfort, revealing views that were emotional and intellectual. Three interlinked sources of discomfort emerged: the perceived eligibility of individual patients or groups for the RCT; the different roles and responsibilities required for RCT participation compared with routine clinical practice; and the tensions between the wish to gain robust evidence and doctors' clinical judgments and personal intervention preferences.

#### 3.2.1 Equipoise and patient eligibility

Doctors were most comfortable when they considered equipoise in general terms, for example, in relation to health-care resources or populations (Box 2). Most also found it relatively easy to approach patients and recruit them to the RCT when they fitted a perceived “core” set of eligibility criteria. Discomfort arose when doctors considered individual patients, or groups of patients, with specific clinical characteristics whom they perceived to be on the “edges” of their perceived core of eligibility. There were considerable differences between doctors in their personal boundary between the core and edges of eligibility, and their boundaries did not always accord with the formal criteria in the RCT protocol. Some doctors had clear views about the most beneficial treatment for edge patients and did not recruit them, whereas others proceeded to recruit, with varying degrees of discomfort depending on their commitment to the RCT (Box 2).

#### 3.2.2 Routine clinical practice, RCT recruitment, and evidence

When doctors compared RCT recruitment with clinical practice, they identified conflicting aspects of their role in decision making with varying degrees of comfort. Recruiting patients to RCTs required them to express uncertainty, and so lack of evidence and equipoise were useful concepts. In routine clinical practice, they had to reach a decision with the individual patient quickly, based on their judgments, often in situations without clear evidence. Doctors varied in terms of which context they found most comfortable (Box 3).

Most doctors were disappointed that evidence was lacking in so many areas, leaving them to rely on instinctive rather than evidence-based clinical judgments. Again, there was evidence of emotion and discomfort, with clinical judgments sometimes referred to somewhat pejoratively as “gut instincts,” “prejudices,” or “hunches”—with doctors committed to the RCTs trying hard to rely on judgments only when they were lacking robust evidence (Box 4).

#### 3.2.3 Equipoise, “hunches,” and intervention preferences

Although these doctors reported relying on community or individual equipoise when recruiting patients, they did not always express personal uncertainty. Some hoped that one treatment (usually their own) might be better than another, and others participated in the RCT because they had clear confidence in their preferred treatment (Box 5). Under the umbrella of community equipoise, some specialists, particularly surgeons, had absolute clarity about the superiority of their own specialty approaches (Box 5).

The degree to which these views impacted on recruitment is considered below.

### 3.3 Equipoise and the fragility of RCT recruitment

All these RCTs were experiencing recruitment difficulties. Although doctors often reported relying on a sense of community equipoise arising from their knowledge of the lack of robust evidence and the existence of a fully funded and approved RCT to justify their participation, many expressed sometimes very strong personal beliefs about the value of particular treatments in general or in relation to specific patient groups. At times, this contradiction caused considerable discomfort, although only a small number thought that their views affected recruitment (Box 6)—the majority cited organizational difficulties, fewer than expected eligible patients, and strong patient treatment preferences [28].

Most seemed able to state that they were “in equipoise” and believe that they could put aside their personal preferences while recruiting. But RCTs T1, T6, and the feasibility phase of T5 had the clearest evidence of strong specialty convictions and the most severe recruitment difficulties. These were RCTs with a surgery arm. Doctors, particularly surgeons, described the conflicts between their sense of equipoise, specialty pride, and personal choice, with some revealing that they really were not in equipoise at all (although they continued to be involved in the RCT; Box 7).

### 3.4 The potential for equipoise to change over time: the role of training and support

The doctors with more experience of taking part in RCTs reported that community equipoise could change over time—for some RCTs to the detriment of recruitment, but others mentioned that a change in the protocol or increased confidence in the RCT could lead to equipoise become more comfortable (Box 8).

In T5, a training and feedback program was developed for recruiters (doctors and nurses) based on the findings of qualitative research integrated into the RCT [29]. Elements of the recruitment intervention were applied to the other five RCTs but in a limited way because of financial and time constraints [19]. In their interviews, the surgeons in T5 described the training and support they had received, the reflection and changes it caused, and their eventual increased levels of confidence and comfort with equipoise and recruitment (Box 9).

## 4 Discussion

This study has shown how doctors, particularly surgeons, experienced and struggled with the concept of equipoise as they carried out recruitment of patients to publicly funded RCTs. None of them espoused a position of theoretical equipoise [9,10]; they were more pragmatic, relying on conceptions of uncertainty at an individual level or, more commonly, on a lack of consensus about specific treatments among colleagues—community equipoise [11,14]. The quest to obtain better evidence to reduce uncertainty was a potent reason for being involved in RCTs and was used frequently as a justification for asking patients to consent to randomization. Many doctors acknowledged that they had “hunches” or “gut instincts” that particular treatments were superior in general or for specific patients or groups, and many experienced discomfort because of their clinical instincts and the “blurring” of equipoise around rigid RCT eligibility criteria. Their discomfort affected their ability and willingness to recruit. Some doctors tried to participate in RCTs even when they had strong convictions about the superiority of their preferred treatment option. They tried to rely on a sense of community equipoise but could not avoid expressing their views or discomfort. Those most committed to RCTs used the need for evidence to suspend their hunches and provide a justification for recruitment, but it seems likely that some would have acted on their hunches, to the detriment of recruitment [28]. Doctors in specialties in which RCTs were common or there was a culture of peer support, such as oncology, tended to find recruitment easier, but there were particular issues in surgery in which clear convictions about their approach, excitement about technical details, and an imperative to practice skills led to RCT recruitment being particularly problematic.

A lack of equipoise among surgeons has been previously identified as a major reason for the small number of successful RCTs in the specialty [30,31], leading to suggestions that other study designs should be used [32]. This study has shown that doctors in a range of specialties can have strong personal preferences, although surgeons did appear to experience particularly intense difficulties in this regard. RCTs in surgery are possible. In T5, for example, where surgery was compared with radiotherapy and no active intervention for localized cancer, surgeons provided with training and feedback learned how to express uncertainty and engage more actively with patients during recruitment [29]. As they became more comfortable with these concepts, recruitment rose from 30% to 65% of eligible patients [33].

The strengths of this study relate to the synthesis of interview findings from doctors actively attempting recruitment across six diverse and challenging RCTs. T2, T3, T4, and T5 eventually completed recruitment with resource extensions and adjusted sample sizes; T1 and T6 closed without completing recruitment. The in-depth nature of the interviews enabled detailed discussion of views and issues, permitting insights that would not have emerged with the use of a questionnaire or structured schedule. Interviews were conducted with surgeons and other clinical specialists who can be particularly difficult to reach. The detailed interview data revealed clear empirical evidence about the conflicts and discomforts experienced by doctors in relation to evidence and equipoise during recruitment and allowed the capture of issues in a range of clinical and RCT contexts, as well as showing the capacity of training and support to bring about changes over time. The limitations include that interviews were carried out with a pragmatic sample of doctors willing to discuss these issues in RCTs already experiencing recruitment difficulties, and so their views may not be representative of others less involved in RCTs or in successfully recruiting RCTs in which there may be more comfort with equipoise. As recruitment is often poor in RCTs, however, it may be that other doctors could have even more adverse views than found here. Interviews were carried out over an extended period because it was important to capture recruiters' views when they were actively recruiting, but we were reliant on RCT CIs being willing to collaborate with the qualitative research and doctors agreeing to be interviewed. This could limit the contemporary applicability of the findings, although recruitment remains a serious difficulty in many current RCTs. Different numbers of doctors were recruited from each RCT because of their scale and individual willingness, and these factors, and that two-thirds of those interviewed were surgeons, may have influenced the findings. Relying only on interview data, rather than including observations or recordings of recruitment appointments, also means that these findings should be considered preliminary. Audio recording appointments proved difficult in five of these six RCTs [19], but the program is gathering these data in ongoing collaborations.

Poor recruitment is repeatedly identified as a serious threat to RCTs [1,4], and this study has elucidated why it is such a fragile process. RCTs should only be mounted following a systematic review of existing evidence and stringent regulation through research ethics and governance procedures [34], but then, the key issues are whether clinicians will commit to recruitment and patients will agree to participate. The concept of clinical equipoise was developed as an ethical justification for carrying out experimental studies on humans [11]. There are strong advocates for its continued use [15] or demise [17,18]. The somewhat esoteric content of the theoretical debate is in sharp contrast with the practical issues raised by the discomfort and emotion expressed by doctors in this study. The quest for evidence was a powerful imperative for these doctors, but they struggled intellectually and emotionally with aspects of equipoise. One solution might be that recruitment could instead be undertaken by nurses, who have been shown to be as effective as doctors [35], or research staff, who might be able to present the RCT more neutrally without clinical responsibility. These suggestions require further research, although there is evidence that nurses' perceptions of their roles can also conflict with recruitment to RCTs, perhaps even more so than doctors [28,36]. Another solution might be to mandate training and support for whoever undertakes recruitment, with audio recording of recruitment appointments to allow scrutiny of the quality of the information they provide to patients, and feedback and support, as in T5 [29]. Rather than agonizing over aspects of the theoretical concept of equipoise, the focus of debate should shift to the practical need to train and support recruiters to engage actively with patients as partners in recruitment [37].

Several findings from this study could be further investigated. Some doctors tried to recruit even when they held very clear opinions about the most effective treatment. Further research should explore whether their preferences can be absorbed within community equipoise [13,38] and whether they can or should recruit patients. The impact of the strong emotion expressed by the doctors in this study, also seen in another qualitative study [39], would also benefit from further development of interventions to assuage it.

As one doctor eloquently put it, “inevitably you can only get patients recruited within the equipoise of the recruiter” (T2-S2). Ultimately, RCT recruitment relies on a recruiter presenting the RCT clearly to potential participants. Doctors in this study, including surgeons, wanted to participate in RCTs to gather high-quality evidence. They tried to rely on a sense of community or individual equipoise but experienced considerable discomfort, intellectually and emotionally, in relation to their clinical and research roles. Methods of training and support need to be developed, as in T5, to enable doctors to gain greater understanding of key RCT concepts so that they can present RCT information with comfort and engage more easily with patients, enabling even the most challenging health-care questions to be addressed through RCTs.

## Figures and Tables

**Table 1:** RCT names, designs, contextual characteristics, and numbers of study participants

RCT code	RCT acronym (see footnotes for details)	RCT type	Clinical centers, *n*	Interventions	Specialties involved	Primary recruiters	Dates of interviews	Doctor interviews, *n*	Follow-up doctor interviews, *n*	Total number of interviews, *n*
T1	EaStER [20]	Feasibility	5	Laser surgery or radiotherapy	Surgery and oncology	Doctors and nurses	2005	3 Surgeons	3 (1 group)	6
T2	FACS [21]	Main	28	Follow-up strategies from low to high intensity	Primary care and oncology	Doctors and nurses	2005	1 GP, 2 surgeons	3 (1 group)	6
T3	PITCH [22]	Main	1 Area (multiple locations)	Ibuprofen or paracetamol or both	Primary care and pediatrics	Nurses	2005	2 GPs	0	2
T4	SWAN [23]	Main	2 Areas (multiple locations)	Support for work or usual care	Psychiatry, community services	Nurses and others	2006	3 Community mental health	2 (1 group)	5
T5	ProtecT [24]	Feasibility and main	9	Surgery or radiotherapy or monitoring	Surgery and oncology	Doctors and nurses	2001/2002, 2006	12 Surgeons, 1 oncologist	7 Surgeons	20
T6	SPARE [25]	Feasibility	22	Chemotherapy with surgery or with radiotherapy	Oncology and surgery	Doctors	2009	4 Oncologists, 4 surgeons	0	8
Total								32	15	47

*Abbreviations:* RCT, randomized controlled trial; GP, general practitioner.

## Box 1 Community equipoise

T6-D4: With T6, like any clinical trial, it's the idea of clinical equipoise, that you have to be able to put your hand on your heart and say that you'd be happy for your patient to get any of the treatments in the trial. You don't think that one of them is worse than the other, and what we're trying to find out is if one is better than the other because we don't currently have the evidence to say one way or the other.

## Box 2 Equipoise and eligibility

T2-D1: I think the uncertainty is about the cost effectiveness of follow up … There's a trade off for the individual to [take part in this RCT] ... For the NHS though the trade-off is the cost of doing this intensive imaging compared to the number of life years gained.

T5-S10: I've not found it [equipoise] personally difficult in concept, because I've been absolutely convinced that we don't know which is the best treatment and although surgery may be a better procedure for cancer cure it has such a high impact that a small number of people might benefit and it's quite a few who are paying a heavy price to get that. So I've not really had any difficulty in that. I think where it's got difficult, I would say is, in a group of patients that intuitively I feel might do better with active treatment … I know intellectually there's no evidence about treatment in particular sub-groups, but ... there's little sub groups where I've found it more difficult than others, and that's inevitable, I think.

## Box 3 Routine practice compared with RCT recruitment

T2-D1: [In clinical practice] I have to make a decision and in the other I don't and that's why I prefer trials … I can hide behind a trial, I don't have to make a decision… I'm doing the best for the patient because I genuinely don't know; we're doing the best for society because hopefully we'll get that answer out.

T5-S4-F: I think that the main difference is that [in clinical practice] I have to steer them to a choice, that there is a specific choice. I can't steer them to equipoise because equipoise is the worst possible outcome for them isn't it? … You give them arguments that build up their feeling about that particular option.

## Box 4 Evidence and clinical “gut” instincts

T6-D4: We don't have the evidence and people's choice of one treatment or another is based predominantly on a sort of prejudice. So if you see an oncologist first, they may be more likely to suggest radiotherapy; if you see a surgeon they may be more likely to suggest surgery.

T2-D1: Yes as a clinician, I'm in equipoise. Yes I know my gut feelings are not to do with my clinical judgment or activity. I don't base my clinical activity on that feeling. I'm very, very distrustful of my gut feeling.

## Box 5 Personal/clinical convictions

T6-D1: I wouldn't be entering patients if I knew what the trial was going to show because I wouldn't have the equipoise required for randomisation. But I'd quite like it to show that organ preservation is at least as good as surgery.

T6-S5: I think the radiotherapists should believe in radiotherapy and I think surgeons believe in surgery. And I think that's probably pretty normal actually. To tell you the truth, I believe in surgery because I'm a surgeon. And I think what we'll find out from the study is whether the radiotherapy treats as well as we do.

T5-S6-F: I don't know, and I believe that that's why I've got equipoise. I don't know. I would say that to them [patients] … I don't know, you don't know, nobody knows … That's why it has to be randomized … Good quality surgery's the best option but I accept that that's not been shown, you know. I accept that it needs to be shown and if you, I think what you're asking me is how do I persuade a patient if I don't believe it myself? … Well, the answer is it's not been shown so it's only a hunch and that's what bias is so I put my biases aside. But if they pin me down and they say, “do you think the earlier you get it the more likely you are to cure it?” Well I've got to say, “yes” because I think that, you know.

## Box 6 The impact on recruitment

T6-S5: I think the younger people with a cancer might be better off having surgery because of the long-term risks of getting a recurrence after radiotherapy and what that means for them in terms of survival. So I probably do have an underlying bias towards surgery. Then again that's what I do so I think as long as you recognise that then if they are deemed eligible then they should all be given all the options. Whether my biases come out, I don't know. I try not to.

## Box 7 The absence of equipoise

T1-S3-F-G: The real thing that swings it for me, if I was faced with this decision I'd have surgery with somebody who would do it properly, for the sole reason that if I have another tumour and I have radiotherapy, it's done and dusted. It gives me multiple treatment options, I can still have more surgery, I can have more extensive surgery and I can still have radiotherapy afterwards and that would be for me personally the key argument.

Interviewer: But then you don't have equipoise personally.

T1-S3-F-G: Umm I don't.

T1-S1-F-G: And you are truly wonderful, [name], but you are still a surgeon, you are still a surgeon and if we had a radiotherapist sitting there they'd say actually in my hands the side effects are very tiny, because they're talking about a small field … they protect things. It is really very minimal.

T5-S8: There's a proportion of patients who will say to me, “What do you think doctor?” And in that situation, I think my gut feeling is important. I always tell them … I wouldn't have become a surgeon if I thought another form of therapy was the best form of therapy, would I? So my preference does matter to some patients but, you know, it's important for them in my opinion to also go and talk to the radiotherapist who, presumably, will be more passionate about their treatment.

## Box 8 Change over time

T4-D1: Well, from the beginning to eighteen months into the trial, the field changed within which the study was taking place and I think we'd underestimated that … Staff have a feeling that to cut out, as they see it, the option of referring to [the intervention] might deprive the patient of something valuable. Now, we don't know if it's a deprivation because we've no idea if this works yet … but in their eyes, it is, you know—I can see why they'd think that.

T6-S5: When I first went into it and the surgeons were saying “oh, well, we don't think so…” and you know, I'm thinking, “should we be doing this?” So I think you do have to get yourself to a point where you feel comfortable with all that's on offer. I mean, quite often when you start a study, you don't always, necessarily … And the more you find out, usually, the more you realize, “well, actually, yeah, this study is pretty important, we don't really know.”

## Box 9 Training and feedback

T5-S7: I found the [training] meeting [about T5] fascinating. Because you don't realise all the prejudices that you do have until you talk to other people about it and you talk about a trial like this and you realise that you are making lots of assumptions, and the main assumption is that as a surgeon you're doing good. Do you know what I mean? If you get rid of something you're doing some good. But you've got to critically evaluate that. It's hard. It's hard to be honest and say, “I'm doing all this work, slaving away and actually, have I achieved anything?”

T5-S9: Becoming slightly more, I hate that word, but in “equipoise”… I try to do that now. I think, I thought I was trying to do that before, but it was a learning process and people point out that using a word like “cure” or the study itself puts treatments in different order, obviously it does change the way you talk to people about it.

T5-S4-F I had reservations [about T5]. I had to think about it for a while before I agreed and certainly in the original meetings about it I was a little bit nervous about how I would cope with dealing with young men with high grade disease etcetera, but the more I thought about it the more sure I am that it is the right thing to do… The whole business is full of a lot of conflicts and, you know, apparent contradictions, but I've got to say that I’ve found myself ultimately comfortable with it all.

T5-S10-F: There has been a remarkable evolution. When I go back to the year 2000, 2001 … Equipoise was not even on the cards. People just could not conceive that men would actually not receive treatment. And over the years, this has changed dramatically and the very people who were so much against equipoise have been recruiting patients because of equipoise.

## References

[1] McDonald A.M., Knight R.C., Campbell M.K., Entwistle V.A., Grant A.M., Cook J.A. (2006). What influences recruitment to randomized controlled trials? A review of trials funded by two UK funding agencies. Trials.

[2] Pighills A., Torgerson D.J., Sheldon T. (2009). Publicity does not increase recruitment to falls prevention trials: the results of two quasi-randomized trials. J Clin Epidemiol.

[3] Brierley G., Richardson R., Torgerson D.J. (2012). Using short information leaflets as recruitment tools did not improve recruitment: a randomized controlled trial. J Clin Epidemiol.

[4] Treweek S., Mitchell E., Pitkethly M., Cook J., Kjeldstrom M., Taskila T. (2010). Strategies to improve recruitment to randomized controlled trials. Cochrane collaboration.

[5] Fayter D., McDaid C., Eastwood A. (2007). A systematic review highlights threats to validity in studies of barriers to cancer trial participation. J Clin Epidemiol.

[6] Kaguelidou F., Amiel P., Blachier A., Iliescu C., Rozé J.-C., Tsimaratos M. (2013). Recruitment in pediatric clinical research was influenced by study characteristics and pediatricians' perceptions: a multicenter survey. J Clin Epidemiol.

[7] Taylor K.M., Margolese R.G., Soskolne C.L. (1984). Physicians' reasons for not entering eligible patients in a randomized clinical trial of surgery for breast cancer. New Engl J Med.

[8] Cheng A.C., Lowe M., Stephens D.P., Currie B.J. (2003). Ethical problems of evaluating a new treatment for melioidosis. Br Med J.

[9] Bradford Hill A. (1963). Medical ethics and controlled trials (Marc Daniels lecture). Br Med J.

[10] Fried C. (1974). Medical experimentation: personal integrity and social policy.

[11] Freedman B. (1987). Equipoise and the ethics of clinical research. New Engl J Med.

[12] Lilford R.J., Jackson J. (1995). Equipoise and the ethics of randomization. J R Soc Med.

[13] Peto R., Biagnet C. (1998). Trials: the next 50 years. Large scale randomized evidence of moderate benefits. Br Med J.

[14] Weijer C., Shapiro S.H., Cranley Glass K. (2000). For and against: clinical equipoise and not the uncertainty principle is the moral underpinning of the randomized controlled trial. Br Med J.

[15] van der Graaf R., van Delden J.J. (2011). Equipoise should be amended, not abandoned. Clin Trials.

[16] Kimmelman J. (2012). The social function of clinical equipoise. Clin Trials.

[17] Miller F.G., Joffe S. (2011). Equipoise and the dilemma of randomized clinical trials. New Engl J Med.

[18] Miller F.G. (2012). Clinical equipoise and risk-benefit assessment. Clin Trials.

[19] De Salis I., Tomlin Z., Toerien M., Donovan J. (2008). Qualitative research to improve RCT recruitment: issues arising in establishing research collaborations. Contemp Clin Trials.

[20] Hamilton DW, de Salis I., Donovan J.L., Birchall M., EaStER: Early Stage glottic cancer: Endoscopic excision or Radiotherapy feasibility study (2013). The recruitment of patients to trials in head and neck cancer: a qualitative study of the EaStER trial of treatments for early laryngeal cancer. Eur Arch Otorhinolaryngol.

[21] Pugh S., Fuller A., Rose P., Perera-Salazar R., Mellor J., George S., Mant D., Primrose J., FACS: Follow-up after Colorectal Surgery (2012). What is the true incidence of metachronous colorectal liver metastases? Evidence from the UK FACS (follow-up after colorectal surgery) trial (ISRCTN: 41458548). Gut.

[22] Hay A.D., Costelloe C., Redmond N.M., Montgomery A.A., Fletcher M., Hollinghurst S., PITCH: Paracetamol plus Ibuprofen for the Treatment of fever in Children (2008). Paracetamol plus ibuprofen for the treatment of fever in children (PITCH): randomised trial. BMJ.

[23] Howard L, de Salis I., Tomlin Z., Thornicroft G., Donovan J.L., SWAN: Supported Work and Needs trial (2009). Why is recruitment to trials difficult? An investigation into recruitment difficulties in an RCT of supported employment for people with severe mental illness. Contemp Clin Trials.

[24] Donovan J.L., Hamdy F.C., Neal D.E., Peters T.J., Oliver S., Brindle L., ProtecT: Prostate testing for cancer and Treatment (2003). Prostate testing for cancer and Treatment (ProtecT) feasibility study. Health Technol Assess.

[25] Paramasivan S., Huddart R., Hall E., Lewis R., Birtle A., Donovan J.L., SPARE: Selective bladder Preservation Against Radical Excision (2011). Key issues in recruitment to randomized controlled trials with very different interventions: a qualitative investigation of recruitment to the SPARE trial. Trials.

[26] Glaser B.G., Strauss A.L., Glaser and Strauss (1967). The discovery of grounded theory.

[27] Miles M.B., Huberman A.M. (1994). Qualitative data analysis.

[28] Donovan J.L., Paramsivan S., de Salis I., Toerien M. (2014). Clear obstacles and hidden challenges: understanding recruiter perspectives in six pragmatic randomised controlled trials. Trials.

[29] Donovan J.L., Lane J.A., Peters T.J., Brindle L., Salter E., Gillatt D., the ProtecT study group (2009). Development of a complex intervention improved randomisation and informed consent in a randomized controlled trial. J Clin Epidemiol.

[30] McCulloch P., Altman D.G., Campbell B., Flum D.R., Galsziou P., Marshall J.C. (2009). No surgical innovation without evaluation: the IDEAL recommendations. Lancet.

[31] Ergina P.L., Cook J.A., Blazeby J.M., Boutron I., Clavien P.-A., Reeves B.C. (2009). Challenges in evaluating surgical innovation. Surgical Innovation and Evaluation 2. Lancet.

[32] Cook J.A., McCullock P., Blazeby J.M., Beard D.J., Marinac-Dabic D., Sedrakyan A., the IDEAL group (2013). IDEAL framework for surgical innovation 3: randomized controlled trials in the assessment stage and evaluations in the long term study stage. BMJ.

[33] Donovan J.L., Mills N., Smith M., Brindle L., Jacoby A., Peters T.J. (2002). Improving design and conduct of randomized trials by embedding them in qualitative research: ProtecT (prostate testing for cancer and treatment) study. BMJ.

[34] Moher D., Hopewell S., Schulz K.F., Montori V., Gøtzsche P.C., Devereaux P.J. (2010). CONSORT 2010 explanation and elaboration: updated guidelines for reporting parallel group randomized trials. BMJ.

[35] Donovan J.L., Peters T.J., Noble S., Powell P., Gillatt D., Oliver S., the ProtecT study group (2003). Who can best recruit to randomized trials? Randomized trial comparing surgeons and nurses recruiting patients to a trial of treatments for localized prostate cancer (the ProtecT study). J Clin Epidemiol.

[36] Tomlin Z., de Salis I., Toerien M., Donovan J.L. (2012). Patient advocacy and patient centredness in participant recruitment to randomized-controlled trials: implications for informed consent. Health Expect.

[37] Truog R.D. (2012). Patients and doctors—evolution of a relationship. New Engl J Med.

[38] Applebaum P.S., Lidz C.W. (2006). Clinical ethics versus clinical research. Am J Bioeth.

[39] Taylor K.M. (1992). Integrating conflicting professional roles: physician participation in randomized controlled trials. Social Sci Med.

