# Supplementary material for: The intellectual challenges and emotional consequences of equipoise contributed to the fragility of recruitment in six randomized controlled trials
Source: J Clin Epidemiol. 2014 Aug;67(8):912–20. doi: 10.1016/j.jclinepi.2014.03.010 (PMC4067744; doi:10.1016/j.jclinepi.2014.03.010)
Supplement: Supplemental Material [file mmc1.pdf]

Intellectual challenges and emotional consequences: doctors’ experiences of equipoise during recruitment of patients in six randomized controlled trials

Web appendix: quotations illustrating key concepts

Included below are additional quotations that illustrate in the doctors’ own words the themes presented in the paper. They are presented below with the same headings or sub-headings as in the paper so that they can easily be linked with the relevant text.

OɸM ‘Community’ equipoise:

T1-S2: If you read the way [expert 1] interprets the data, then there’s a clear advantage for surgery, whereas if you actually read the way [expert 2] interprets the data then there’s a clear advantage for radiotherapy. So that’s why foremost the trial needs doing.

OɸHɸM Equipoise and patient eligibility

T2-S2: I think that actually we are doctors first. If clearly persuading a 95 year old to go in a trial that might involve coming up every six months for a CT scan, I mean that’s ridiculous. You’re a doctor first and it’s your patient so you, you know, you’ve got to pass those hurdles, but then the vast majority of patients you can actually honestly say ‘well I don’t know what would be best for them’.

T5-S11-F: Well I think you can define it [equipoise] when you are looking at populations. I think it’s easy to define equipoise … that I genuinely don’t know and I honestly don’t think that I have a hunch that one treatment’s the best ... It’s on the edges that it [equipoise] begins to break. Yes, it has to be and it should do as well because actually that’s the point of the trial. It’s addressing a question for a particular group of patients, so inevitably, you know, there are blurry bits around the edges where you think you know what’s best ... So for populations it’s easy and again for some individuals it would be easy, but there’s a spectrum isn’t there?

OɸHɸH Routine clinical practice, RCT recruitment and evidence

T5-S10: [In clinical practice] I guess that subconsciously I would have manoeuvred most of them towards surgery, and you can do that, however you say things, whichever words you choose.

T1-S1: In my clinical practice for the vast majority of patients I still have to go by instinct but it’s an instinct that I am continually trying to hone ... I go to a lot of conferences read a lot in the subject and my practice is continually changing in light of the evidence that I accumulate ... but for most of what we do there is not the evidence and therefore you still have to go by gut instinct.

OɸHɸO Equipoise, ‘hunches’ and intervention preferences

T1-S1: If [patients] say, ‘I don’t want radiotherapy because it means trundling up to the radiotherapy centre’, now part of me at that point as a surgeon - um clearly it’s important to be honest here – I would say ‘hoorah, [laughs] these are really good operations, these are really quite good fun’.

T5-S4-F: The hardest thing for a surgeon to do is to do nothing, even though it’s the right thing to do, and I have more agonies about sitting on my hands. You know, surgeons aren’t naturally lazy. You know they always want to do something and it might not be the right thing to do but if you think this patient’s going to come to grief you prefer that they come to grief with you having tried than done nothing, so, so not doing surgery is a hard option to follow for a for a surgeon. It’s hard at an emotional level but not at an intellectual level.

0◊0 Equipoise and the fragility of RCT recruitment

T6-D2: I think some of the surgeons still are putting patients into T6 because they ‘know’ quote-unquote, that surgery will come out on top. I don’t have an issue with that as long as they’re able to discuss the study in a balanced fashion rather than giving any subconscious message from verbal or nonverbal cues that, you know, surgery is the right thing to do. If the reason why they’re putting patients into the study is because they know that surgery is going to be better, that’s fine as long as that’s not what’s coming across to the patient, because then that’s going to deter recruitment.

T1-S2: It is widely believed that you can’t do a randomized trial in head and neck surgery full stop and particularly this, because it’s too difficult. Surgeons know best and surgery is clearly so much better then radiotherapy ... Although the radiotherapists of course think the radiotherapy is best.

T6-S5: The trials have basically shown that the patients do do slightly better with surgery and the trials that have shown equivalence to radiotherapy have been patients who have recurrence after radiotherapy then went on to have surgery anyway ... So, I think it’s difficult for patients to really believe that surgery isn’t a better treatment.

0◊п The potential for equipoise to change over time: the role of training and support

T2-D1: They [surgeons] are not, you know, this word equipoise - they’re not in equipoise about this trial and whether [making a change to the protocol] will bring more surgeons into equipoise about feeling comfortable about offering a trial like this to their patients, only time will tell.

T5-S2-F: Having done this study, gaining this valuable experience of explaining the randomisation and seeing the patients that are, after all this counselling, truly they have come to the idea of randomisation... It used to be not the thing for a surgeon in the beginning to say, uncertainty, and in fact I had a phase where I was very uncomfortable... But increasingly I have now become much more open… I say, ‘I do not know. I wish I had the answers, but no, I don’t.’ So being undecided used to be an uncomfortable feeling, but now, yes, it’s become part and parcel of it.
